# GSP Cochlea: A graph signal processing approach for studying sound encoding

**DOI:** 10.1093/pnasnexus/pgag134

**Published:** 2026-04-21

**Authors:** Melia E Bonomo, Santiago Segarra, Robert M Raphael

**Affiliations:** Department of Physics and Astronomy, Rice University, Houston, TX 77005, USA; Department of Electrical and Computer Engineering, Rice University, Houston, TX 77005, USA; Department of Bioengineering, Rice University, Houston, TX 77005, USA

**Keywords:** cochlea, complex systems, graph signal processing, graph theory, hearing loss

## Abstract

Humans are able to hear in a variety of complicated acoustic environments. This feat begins in the peripheral auditory system, where the cochlea collects and transmits thousands of individual bits of sound data to the brain. Here, we introduce GSP Cochlea: a graph signal processing-based framework to investigate and visualize sound encoding. We show that a cochlea graph with a mesh topology provides a mechanism of denoising, efficient information transfer, and modular processing. We demonstrate an application to assess hearing loss as more than just a decibel loss at particular frequencies of sound but also a significant change to the cochlea graph architecture. GSP Cochlea is a generalized approach that provides new insight into the higher-level functional activity of the inner ear.

## Introduction

The cochlea is a spiral cavity in the inner ear that performs a time-frequency decomposition of sound waves and relays that information to the brain (1, 2). The sound encoding process relies on two types of sensory cells: outer hair cells (OHCs), which serve as amplifiers, and inner hair cells (IHCs), which detect the individual frequency components of a sound wave. The human cochlea contains about 12,000 OHCs and 3,500 IHCs that encode frequencies from ∼20 Hz to 20 kHz. Acoustic vibrations due to an incoming sound cause mechano-sensitive ion channels on these cells to open. An influx of potassium ions depolarizes the cell and opens voltage-gated calcium channels that mediate the release of neurotransmitters onto afferent nerve fibers. Often compared with a piano keyboard, local stimulation of hair cells follows a tonotopic mapping: high-frequency components of the incoming sound cause peak activity at the base of the cochlea and low-frequency components stimulate the apex.

Well-established computational models take a classical signal processing approach and decompose the whole-cochlea response into activity profiles for individual cells and fibers (3). While these simulations provide valuable insight into the processing of individual stimuli, there is currently no method for distilling responses to multiple stimuli into a single visualization or for studying the overarching functional relationships between cells. Additionally, the field has yet to uncover any higher-level organization in the cochlea that may facilitate the simultaneous encoding, denoising, and transmission of such a large amount of sound data to the central auditory system.

Graph theory (4) and, more recently, graph signal processing (5) have had great success in discovering higher-level functional properties in complex biological systems. Machine learning techniques have emerged to infer the underlying topology of biological graphs for which the links cannot be explicitly observed (6). Graph-based methods have not yet been developed to study the ear. In this brief report, we introduce “GSP Cochlea,” which is a generalized framework to characterize, visualize, and analyze cochlear function and dysfunction using graph signal processing (GSP).

## GSP Cochlea formulation

**Figure 1:**
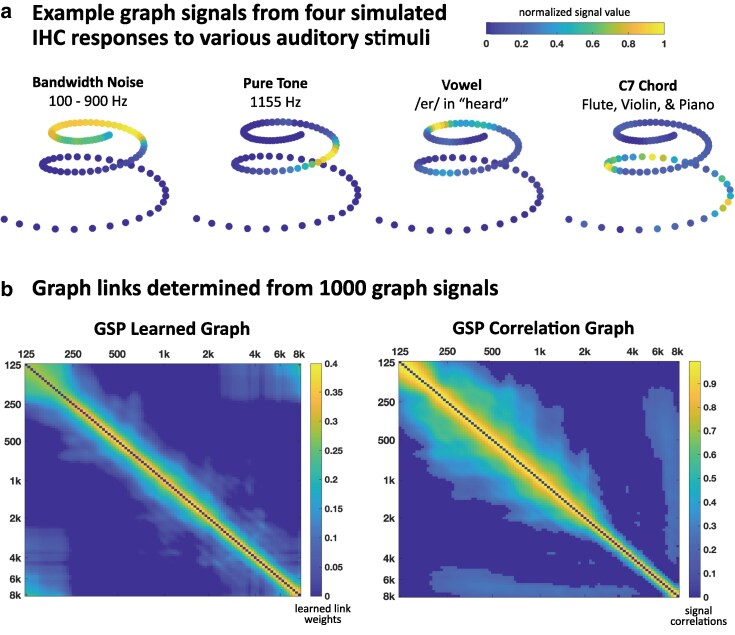
GSP Cochlea formulation. a) Graph signals are generated on 100 nodes from the simulated responses of IHCs. b) GSP Cochlea utilizes 1,000 stimuli to determine functional graph links and their weights, either through machine learning or the pairwise correlations between signals. Matrix nodes are labeled according to their characteristic frequencies (Hz).

The components of sound encoding can be intuitively broken down into elements of a graph. Here, we use IHCs to represent physical nodes. Each node is assigned a performance factor representing the degree of hearing loss, if any. We use the UR_EAR toolbox (3, 7) to simulate an IHC's voltage response when stimulated by an incoming sound, and we define this response as the node's graph signal. Coordinates from a three-dimensional (3D) model of the human cochlear spiral (8) are extracted to visualize each node's anatomical position (Fig. 1a, SI Appendix). The graph links are the functional relationships between cells associated with how the whole cochlea responds to incoming sounds.

We investigate four different methods of determining graph links (SI Appendix): (i) GSP learned graph: We assume the values of the graph signals change smoothly between connected nodes and conduct network inference using the GSPBOX toolbox (9, 10). (ii) GSP correlation graph: We calculate the correlation coefficient between the signals of each pair of nodes. While a correlation graph is not exclusive to GSP, we include it in the GSP category given that it makes use of graph signals. Figure 1b shows the connectivity matrices for these two GSP-based graphs. The final two methods do not use GSP principles or observations of IHC responses. (iii) Frequency graph: We calculate the inverse of the characteristic frequency difference between each pair of nodes. (iv) Linear graph: We follow a traditional auditory processing paradigm and link nodes that are anatomically adjacent in a line topology.

**Figure 2:**
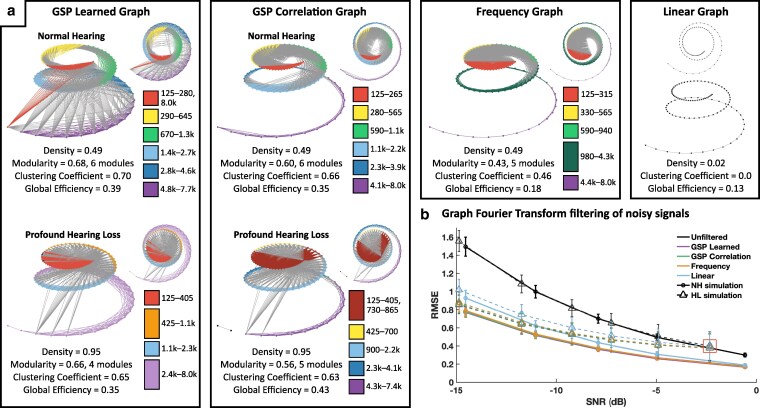
Analysis of graph structure. a) GSP-based graphs are created for normal hearing (NH) and for a patient with hearing loss (HL) using their specific audiogram. The key next to each graph shows the characteristic frequencies (Hz) in each module. Graph nodes and links are color-coded according to their module allegiance. For the mesh graphs, only the links at 0.25 density are displayed. b) GFT filtering is performed using each graph structure on five sets of 1,000 signals for which we altered the SNR. Given that the graph signals with HL already contain a baseline level of −2.3 dB noise when compared with their NH counterparts, the red square indicates a sixth set of signals with no additional noise added. Performance is measured using the root mean squared error (RMSE) between the original and graph filtered signals. Error bars represent SD. Two-sample t tests were calculated between each pair of curves at each SNR tested.

We then analyze the organization and performance of the resulting cochlea graphs (Fig. 2, SI Appendix). First, we use modularity to measure the degree to which there are communities of tightly interacting nodes (11). The GSP-based graphs both have relatively high modularity, compared with the frequency graph. Next, we examine the information processing capabilities. We find that both GSP-based graphs have higher clustering and global efficiency than the frequency graph, and all three of these mesh graphs had higher metrics than the linear graph. Finally, we perform graph Fourier transform (GFT) filtering with GSPBOX to test how well each graph can denoise cochlear signals. Across all signal-to-noise ratios (SNRs) tested, the mesh graphs perform significantly better than the line graph (P<0.0001), especially as noise increases. At SNR worse than −5 dB, both GSP-based graphs perform slightly better than the Frequency graph (P<0.0001).

## Using GSP Cochlea to study hearing loss

Audiograms from 224 patients in the AudGenDB dataset (12) are utilized to calibrate the performance of each IHC in UR_EAR, and the GSP learned method is used to generate a cochlea graph for each patient (SI Appendix). With increasing hearing loss severity, there is a strong positive correlation with graph density (r=0.93,P<0.0001) and moderately negative correlations between hearing loss severity and modularity (r=−0.31,P<0.0001), the number of modules (r=−0.32,  P<0.0001), global efficiency (r=−0.38,P<0.0001), and average clustering (r=−0.57,P<0.0001). There are no relationships between these graph measures and age, sex, race, or ethnicity.

We then perform the GFT filtering analysis. A patient with profound hearing loss is randomly selected, and we generate a GSP learned and a GSP correlation graph using their audiogram (Fig. 2a). Across all SNRs tested (Fig. 2b), both GSP-based graphs are better at filtering signals than the linear graph (P<0.0001). The GSP-based graphs are slightly better than the frequency graph: GSP learned is better when SNR is worse than −9 dB (P<0.016), and GSP correlation is better when SNR is worse than −12 dB (P<0.013). For all graphs, however, the SDs are much larger than those for normal hearing, pointing to unreliable noise filtering.

## Discussion

In comparison to the traditional linear paradigm, mesh graphs consisting of cross-cochlea functional relationships appear to be better poised to process incoming sound data. In complex systems, clustering and global efficiency promote segregated and integrated information processing, respectively, and modularity confers robustness and adaptability (4). These graph features, which are most prominent for normal hearing and patients with little to no hearing loss, could help the cochlea with encoding pitch and timbre information, as well as with the general processing demands introduced by such a wide variety of stimuli. Furthermore, given that brain activity leverages a mesh topology and modular organization, it would be practical for data coming from the periphery to be similarly structured. While mesh graphs are better at denoising cochlear signals, the connection between GFT filtering and a biological mechanism of action remains to be determined.

GSP-based methods can augment the study of hearing loss by generating patient-specific cochlea graphs. Currently, audiograms are the standard metric for diagnosing hearing loss. However, deficits in cross-cochlea functional connections, lower modularity, and disruption of information flow could account for the difficulties that patients face beyond what is measured in an audiogram, such as speech perception in noisy environments or music listening.

There are a couple of limitations with our example GSP Cochlea formulation. First, the IHC responses are obtained from a simulation, meaning that our cochlea graphs reflect the specific physiological features that are coded into UR_EAR. The GSP Cochlea framework is certainly amenable to other phenomenological or biophysical models (3), and further investigation could determine the extent to which any graph features depend on a particular model of monaural processing. Second, the graph signal values are generated by averaging over the IHC's time-series response, which means that temporal information is not being used in the generation of the graph. Future work could utilize the full time series to explore time-varying graphs of sound encoding.

We have demonstrated how sound encoding can be visualized and studied as a 3D cochlea graph. GSP Cochlea is a highly flexible framework that can be expanded to include additional levels of complexity for studying data representations and flow between the ear, midbrain, and auditory cortex. For example, with the inclusion of auditory nerve fibers as nodes, neural fluctuations (13) could be used as the graph signals. Further probing how graph structure impacts the cochlea's function may shed light on other phenomena, such as otoacoustic emissions (14). In clinical applications, a patient's personal cochlea graph could be used to enhance individualized device settings by incorporating their graph into the classical signal processing strategies of hearing aids and cochlear implants.

## Supplementary Material

pgag134_Supplementary_Data

## Data Availability

Computer codes written for the GSP Cochlea formulation presented here are available at https://github.com/meliabonomo/GSPcochlea.
