# Supplementary material for: GSP Cochlea: A graph signal processing approach for studying sound encoding
Source: PNAS Nexus. 2026 Apr 21;5(5):pgag134. doi: 10.1093/pnasnexus/pgag134 (PMC13148644; doi:10.1093/pnasnexus/pgag134)
Supplement: pgag134_Supplementary_Data [file pgag134_supplementary_data.pdf]

PNAS nexus

Supplementary Information for
GSP Cochlea: A graph signal processing approach for studying sound encoding

Melia E. Bonomo[1]*, Santiago Segarra[2], Robert M. Raphael[3]
1. Department of Physics and Astronomy, Rice University, Houston, TX 77005, USA
2. Department of Electrical and Computer Engineering, Rice University, Houston, TX 77005, USA
3. Department of Bioengineering, Rice University, Houston, TX 77005, USA

* To whom correspondence should be addressed.
Email: mbonomo@rice.edu

This file includes:

Extended Methods
SI References

Extended Methods

GSP Cochlea Formulation Summary. For the example formulation of GSP Cochlea presented in this Brief Report, we consider a graph with 100 nodes to represent hubs of about 40 IHCs, which is comparable to the frequency resolution of cochlear implants. Each IHC hub is labeled with a characteristic frequency of excitation from 125 Hz to 8 kHz based on its anatomical position (see **Graph Visualization** below). Initially, we assume all nodes are functioning perfectly, i.e., without any hearing loss. We sample 1000 stimuli evenly distributed across the range of IHC characteristic frequencies from a set of pure tones, musical notes and chords, vowels, and bandwidth noise (see **Auditory Stimuli** below).

Each stimulus is run through the University of Rochester: Envisioning Auditory Responses (UR_EAR) MATLAB toolbox [**1**] to simulate IHC voltage activity. Briefly, UR_EAR is a phenomenological model developed over several decades that simulates each step of monaural processing using sub-models for the middle ear, cochlear filtering, inner hair cell, auditory nerve synapse, cochlear nucleus, and inferior colliculus. During the cochlear filtering stage, UR_EAR contains two parallel processing paths: a static filter bank and a dynamic one that adapts based on broad-band filters representing outer hair cell gain-control. IHC sensitivities are tonotopically coded (see **Graph Visualization** below), and their voltages are determined from a nonlinear transformation of the cochlear filter bank output. The filters are tuned to a human cochlea based on experimental measurements, and there is a mechanism to estimate outer and inner hair cell impairment based on a given audiogram.

We average and normalize the voltage response of each IHC hub for each stimulus, yielding 1000 graph signals. Four different methods of determining graph edges are investigated (see **Graph Edges** below). We then utilize real patient audiograms to configure each node's performance factor and explore the impact of hearing loss. We utilize audiograms from 224 patients in the AudGenDB dataset [**2**] and UR_EAR to calibrate the performance factor of each IHC graph node (see **Audiogram Data** below). There are seven levels of hearing loss severity, with 32 patients in each of these diagnosis groups: normal (i.e., no hearing loss), slight, mild, moderate, moderately severe, severe, and profound. We generate graph signals from 1000 unique stimuli for each patient. Our initial analysis of the GSP Learned and GSP Correlation graphs of normal hearing had comparable graph theory metrics, indicating that the signals can be used in more than one way to determine graph edges. We therefore chose to focus on one method, GSP Learned, to determine graphs for all patients and generate an additional patient GSP Correlation graph for GFT filtering.

Graph Signal Processing (GSP). Let us consider a graph $G(V, E, \mathbf{W})$, where V is a set of M nodes $\{v_1, \ldots, v_M\}$, E is a set of unordered pairs $\{(v_i, v_j) \ldots\}$ representing edges (i.e., links) between nodes, and $\mathbf{W}$ is a connectivity matrix containing the weights W_{ij} of each edge $(v_i, v_j) \in E$ [**3**]. Each node v_i is a sensory unit in the cochlea labeled with the characteristic frequency f_i to which it exhibits a peak response. The E and $\mathbf{W}$ represent functional relationships and their strength between physical nodes (see **Graph Edges** below). We define the vector $\mathbf{x}_k = [x_1, \ldots, x_M]^\top$ as a signal residing on G, where x_i is the value associated with node v_i. The signal is the cochlear response to a particular sound stimulus k, and this response is impacted by how well the sensory units are functioning. Thus, each node v_i has an associated performance factor $0 \leq b_i \leq 1$, where 1 means the node is functioning perfectly and 0 means the function is severely impaired. If the cochlea response to a stimulus is measured as a time series, we average the time series for each node and then normalize across all nodes to obtain the signal vector $\mathbf{x}_k$. This is repeated for each stimulus to obtain all N graph signals. A set of N signals on M nodes yields the matrix $\mathbf{X} \in \mathbb{R}^{M \times N} = [\mathbf{x}_1, \ldots, \mathbf{x}_N]$, where each row of signal values $X_i \in \mathbb{R}^N$ resides on a node v_i.

Graph Edges. We evaluate four methods of determining graph edges. The first three methods by design yield graphs with mesh topologies (i.e., edges between non-adjacent nodes) that all have equal degrees of freedom (i.e., edge density), and the fourth method tests a null graph model by connecting nodes with a line topology.

1. GSP Learned Graph: Machine learning on smooth graph signals.
The $\mathbf{W}$ matrix (and therefore E) is learned by assuming the graph signals are smooth across connected nodes [**4**]. A pairwise distance matrix $\mathbf{Z} \in \mathbb{R}^{M \times M}$ is computed between each row of the

signal matrix $\mathbf{X}$, as $Z_{ij} = \| X_i - X_j \|^2$, and put into the GSPBOX MATLAB toolbox [**5**] to learn graph edges using

$$\min_{\mathbf{W}\in W_m} \|\mathbf{W} \circ \mathbf{Z}\|_{1,1} + y(\mathbf{W}),$$

where W_m is the space of all valid weighted adjacency matrices $\mathbf{W}$, and $y(\mathbf{W})$ is a function that prevents the adjacency matrix from going to the trivial solution $\mathbf{W} = 0$ while promoting sparsity (i.e., some zero edges) [**4**]. The method used in GSPBOX is

$$y(\mathbf{W}) = -\alpha \mathbf{1}^{\top} \log(\boldsymbol{W}\mathbf{1}) + \frac{\beta}{2} \|\mathbf{W}\|_F^2,$$

where $\mathbf{1} = [1, \ldots, 1]^{\top}$ and $\|\cdot\|_F$ is the Frobenius norm [**4**,**5**]. The first term imposes a logarithmic barrier that penalizes the formation of disconnected nodes, such that individual edges may be zero but the degrees must be positive. Increasing α allows for larger edge weights, but on its own (i.e., when β is zero), it does not impact the sparsity of the connectivity matrix. The second term penalizes the formation of very large weights but allows smaller weights to flourish. Increasing β thus allows for more edges and therefore denser connectivity matrices. Given the novelty of using graph theory to study auditory processing, there is no established precedent for the optimal α and β values. For this reason, we set these parameters *ad hoc*. The GSPBOX toolbox demonstrates that general graph learning from smooth signal data can be implemented with

$$s = \frac{1}{2} \sqrt{2(M-1)} \,/3,$$

where $\alpha = 2s$ and $\beta = s$ [**6**]. We keep this parameter s constant for learning the general cochlea graph as well as each individual patient graph so that the network measures across all graphs are comparable. Furthermore, as described in sections 2 and 3 below, we create two additional cochlea graphs that are not determined via machine learning and are thresholded to the same edge density as the learned graph to control for this parameter.

In the learned weighted adjacency matrices, an edge (v_i, v_j) means that for any sound stimulus coming into the cochlea, the values of the response signal of nodes v_i and v_j will be similar, according to the strength of the edge weight W_{ij}. No edge would mean the signal values of nodes v_i and v_j are usually very different.

2. GSP Correlation Graph: Correlations between graph signal values.
All IHC nodes are initially fully connected. The Pearson correlation coefficient is calculated between the graph signal values $X_i \in \mathbb{R}^N$ of each pair of nodes as

$$\rho(X_i, X_j) = \frac{\operatorname{cov}(X_i, X_j)}{\sigma_i \sigma_j},$$

where $\operatorname{cov}(A, B)$ is the covariance of variables A and B, and σ_A is the standard deviation of A. This yields a pairwise correlation matrix $\mathbf{W} \in \mathbb{R}^{M\times M}$, where each edge weight is $W_{ij} = \rho(X_i, X_j)$. The graph is then thresholded to have the same number of edges as the GSP Learned graph.

The purpose of including the GSP Correlation graph as a benchmark is to evaluate the benefit of promoting smoothness in the graph structure via an optimization framework (as is done in the GSP Learned graph), compared to simply looking at the correlations between signals. Though a correlation graph is not exclusively a GSP method, we categorize it as such to distinguish it from the methods that do not make use of graph signals in the generation of the graph topology (i.e., the Frequency and Linear graphs).

3. Frequency Graph: Differences between characteristic frequencies.
All IHC nodes are initially fully connected. The inverse of the difference between the characteristic frequencies f_i of each pair of nodes is calculated to obtain edge weights. This graph is also thresholded to have the same number of edges as the GSP Learned Graph. The purpose of including the Frequency graph as a benchmark is to evaluate a mesh topology in a graph that was not devised using observed signals and GSP principles, but rather is formulated *a priori* from each node's characteristic frequency.

4. Linear Graph: Connections between anatomically adjacent nodes.
An edge is drawn between each pair of anatomically adjacent IHC nodes, resulting in $M - 1$ edges. Though the edge density is substantially different than the other three graphs, the purpose of including this Linear graph is to evaluate the traditional paradigm that auditory processing is carried out by a line configuration of cells along the cochlear spiral.

Graph Visualization. To visualize our cochlea graphs, we extracted anatomical dimensions and coordinates of the spiral from a 3D scaling model of the human cochlea in MATLAB [**7**]. The 3D model was developed from corrosion cast imaging and micro-CT scanning of 138 human cochleae, which showed a mean length of the lateral outer wall to be 40.9 ± 2.0 mm and a mean number of turns to be 2.68 ± 0.12 [**7**]. To position each inner hair cell (IHC) node, we used the Greenwood function, which is a species-dependent equation that describes the relationship between position of an inner hair cell along the spiral and its characteristic frequency [**8**]. The equation for characteristic frequency f_i in Hz of node v_i based on position r_i in mm in humans is

$$f_i = 165.4\,(10^{0.06 r_i} - 0.88).$$

Though recent work in guinea pigs finds a deviation from this traditional mapping at low frequencies [**9**], Greenwood mapping was nonetheless used in the example formulation of GSP Cochlea presented here to match up with the tonotopy coded into the UR_EAR MATLAB toolbox [**1**]. UR_EAR utilizes [**8**] for parameterizing the center frequency of IHC filters based on the location on the human basilar membrane and [**10**] for tuning sharpness in humans.

Graph Analytics. Statistical properties were calculated to quantitatively compare the structure across different cochlea graphs. The Python NetworkX package [**11**] was used to calculate various graph-theoretic measures: graph density, average clustering, global efficiency, and modularity. Graph density is the percentage of connected edges calculated as the number of edges that exist divided by the total possible edges that the graph could support. In a graph with 100 nodes, the total possible number of edges if each node was connected to every other node is 4,950. For average clustering, global efficiency, and modularity, all graphs are binarized to keep the top edges at 0.10 density so that these measures, which depend on the number of edges, can be compared across graphs that otherwise have different densities. Clustering of a node is the number of edges between a node's neighbors divided by the node's total edges, and this is averaged for all nodes to obtain the average clustering. Efficiency of a pair of nodes is the inverse of the shortest path length between the nodes, and since the graphs are weighted, the shortest path length is calculated using the Dijkstra algorithm and the inverse of edge weights. The inverse weights are used such that efficiency will reflect shortest paths along the strongest weights. Global efficiency is then the average efficiency of all pairs of nodes. Graph modularity is the degree to which there are communities of tightly interacting nodes, defined as

$$Q(\{\phi\}) = \frac{1}{2D} \sum_k \sum_{ij \in \phi_k} \left(A_{ij} - \frac{d_i d_j}{2D} \right),$$

where D is the total number of edges, d_i is the degree of node i, $\mathbf{A}$ is the binarized connectivity matrix, and ϕ_k is module k [**12**]. Q is calculated based on a particular configuration of nodes into modules, $\{\phi\}$, as the number of within module edges divided by the total number of edges in the graph, scaled by the probability of finding an edge between nodes inside the same module in a random graph with the same degree distribution. Modularity was determined by finding the partition of nodes into modules that maximizes Q using the Python Modularity Maximization package [**13**]. The modularity maximization algorithm randomly assigns a module label, and therefore to compare modules across different patient graphs, we utilized a super-module technique previously described in [**14**]. Briefly, all of the modules found across all 224 patient cochlea graphs are now considered "super"-nodes, and the Jaccard index between all pairs of modules is calculated to yield the edges between these super-nodes. For example, two modules found in different patient graphs that have a high similarity in their node membership would have a strong edge; whereas two modules found in the same patient graph will have no overlap in their node membership, and therefore no edge. Modularity maximization is then run on this "super" graph, and the super-modules that are found contain all of the individual modules from the 224 patient graphs that have comparable node memberships.

Graph Fourier Transform (GFT) Filtering. The four graphs were tested on how well they could each filter noisy signals using GSPBOX [**5**]. Five new sets of N=1000 stimuli were sampled; these were clean stimuli that did not overlap with the stimuli that had been used to generate the graph signals for the GSP Learned and Correlation Graphs. The stimuli were run through the UR_EAR MATLAB toolbox [**1**], calibrated for normal hearing, to simulate voltage response timeseries for each of 100 IHCs, matching up to the 100 nodes in the GSP Cochlea and linear graphs. The voltage timeseries were averaged and normalized to generate a clean ground truth graph signal for each stimulus, resulting in the matrix $\mathbf{X} \in \mathbb{R}^{M\times N}$ for each set of stimuli. We then added Gaussian noise $\mathcal{N}(0, \sigma^2)$ to these graph signals, each set of 1000 stimuli receiving noise with a different standard deviation, $\sigma = 0.3, 0.5, 0.7, 1, 1.5$. The signal-to-noise ratio (SNR) was then calculated between each original signal, $\mathbf{x}$, and noisy signal, $\tilde{\mathbf{x}}$,

$$\mathrm{SNR} = 10\log_{10}\left(\frac{\sum_{i=1}^{M} x_i^2}{\sum_{i=1}^{M}(x_i - \tilde{x}_i)^2}\right),$$

as was the root-mean-squared-error (RMSE) to show the error in the noisy, unfiltered signal

$$\mathrm{RMSE} = \sqrt{\frac{\sum_{i=1}^{M}(x_i - \tilde{x}_i)^2}{M}}.$$

RMSE was averaged over all N signals for a particular set. We then used GSPBOX to compute a GFT of each signal with respect to the Fourier basis of each graph, G. The graph Fourier multipliers were approximated using shifted Chebyshev polynomials. GFT filtering was then performed on the noisy signals using a first order low pass filter in the spectral domain $h(\lambda) = 1/(1+\lambda)$ and the normalized Laplacian of each graph as the shift operator. Using the normalized Laplacian enforced an upper bound on the maximum eigenvalues, such that for all graphs the eigenvalues were on the interval [0 2]. The RMSE was calculated between each resulting signal, $h(\tilde{\mathbf{x}})^G$, filtered by one of the four graphs tested, and the original signal, $\mathbf{x}$,

$$\mathrm{RMSE} = \sqrt{\frac{\sum_{i=1}^{M}(x_i - h(\tilde{x})_i^G)^2}{M}}$$

and averaged over all N signals for each stimulus set.

The same general procedure as described above was followed to study how graphs with hearing loss (HL) performed. The UR_EAR was first calibrated using the audiogram of a patient with profound hearing loss (see **Audiogram Data** below). The same five sets of N=1000 stimuli were gathered and put through the UR_EAR-HL to obtain graph signals. We added varying amounts of Gaussian noise to the output signals. It is important to note that the raw signals coming out of the UR_EAR-HL, $\mathbf{x}^{\mathrm{HL}}$, are already "noisy" compared to the UR_EAR with normal hearing (NH), with an average SNR of -2.3 dB. Given this, a sixth set of N=1000 graph signals were generated from UR_EAR-HL and no additional Gaussian noise was added. The standard deviations of added noise were therefore $\sigma = 0, 0.3, 0.5, 0.7, 1, 1.5$. The signals generated by UR_EAR with NH were considered the ground truth "clean" signals, so SNR and RMSE of the unfiltered UR_EAR-HL output with added noise, $\tilde{\mathbf{x}}^{\mathrm{HL}}$, were calculated with respect to $\mathbf{x}$,

$$\mathrm{SNR} = 10\log_{10}\left(\frac{\sum_{i=1}^{M} x_i^2}{\sum_{i=1}^{M}(x_i - \tilde{x}_i^{\mathrm{HL}})^2}\right),$$

$$\mathrm{RMSE} = \sqrt{\frac{\sum_{i=1}^{M}(x_i - \tilde{x}_i^{\mathrm{HL}})^2}{M}},$$

and RMSE of the noisy signal after filtering, $h(\tilde{\mathbf{x}}^{\mathrm{HL}})^G$, was calculated with respect to $\mathbf{x}$

$$\mathrm{RMSE} = \sqrt{\frac{\sum_{i=1}^{M}(x_i - h(\tilde{x}^{\mathrm{HL}})_i^G)^2}{M}},$$

and averaged over all N signals for each stimulus set. Two-sample t-tests were performed in MATLAB between RMSE results for all pairs of graphs.

Auditory Stimuli. A dataset of 14,934 stimuli was generated, which included pure tones, musical notes and chords, vowels, and bandwidth noise. All stimuli were a maximum of 0.5 s (vowels were shorter), and they were set to the same sound pressure level (65 dB) and sampling rate (100 kHz). Pure tones from 125 Hz to 8 kHz at 5 Hz intervals (1,576 total) were created with a 0.02 s ramp duration using the UR_EAR MATLAB toolbox [**1**]. Individual musical notes were downloaded from the Musical Instrument Samples dataset for flute (39 notes, 247 Hz to 2.2 kHz), trumpet (36 notes, 165 Hz to 1.1 kHz), violin (92 notes across 4 strings, 196 Hz to 2.6 kHz), and piano (61 notes, 130 Hz to 4.2 kHz) [**15**]. These musical samples were cropped to 0.5 s and provided 228 single pitch, single timbre stimuli. We combined two notes from a particular instrument into new files for major (4-note spacing) and minor (3-note spacing) chords, yielding 407 multi pitch, single timbre stimuli. We then combined three notes from different instruments into new files for major (4-3-note spacing) and minor (3-4-note spacing) chords, yielding 10,152 multi pitch, multi timbre stimuli. Vowels (1668 total) from 139 speakers (coded as man, woman, boy, or girl) were acquired from the Hillenbrand dataset [**16**]. Gaussian noise stimuli were created with a 0.02 s ramp duration using UR_EAR from 100 Hz to 8 kHz at various bandwidths: 50 Hz (158 stimuli), 100 Hz (157 stimuli), 200 Hz (155 stimuli), 400 Hz (151 stimuli), 800 Hz (143 stimuli), 1 kHz (139 stimuli). The large number of real-world stimuli (musical notes, chords, and vowels) meant that the bulk of the stimuli in our dataset had fundamental frequencies F0 (or F1, in the case of the vowels) between 200 Hz and 2 kHz. Therefore, when sets of 1,000 stimuli were randomly sampled to generate graph signals, careful attention was paid to sample stimuli evenly across the frequency spectrum of our graph nodes (125 Hz to 8 kHz). This ensured that all IHCs would be sufficiently stimulated and minimize any potential boundary effects in our learned graph edges.

Audiogram Data. To study the impact of hearing loss, we used audiogram data from the AudGenDB Postgres 12 archive [**2**]. An audiogram reports the sound pressure level (in dB) needed for a patient to hear a particular frequency, so the test can reveal insufficient sensitivities. The audiogram as a whole is coded by the medical provider with an overall level of hearing loss severity: normal, slight, mild, moderate, moderately severe, severe, and profound. In AudGenDB, a patient file may contain multiple visits with the medical provider and even multiple audiograms performed during a single visit (e.g., in different ears or test conditions), therefore an individual patient may have multiple levels of hearing loss severity coded into their file for different ears and/or time points. For the purposes of our analysis, we consider each unique audiogram and hearing loss severity pair to be an individual "patient" (hereafter referred to as such). We randomly sampled 32 individual patients from each hearing loss severity group in AudGenDB, resulting in 224 pediatric patients. To do so, we first excluded patients who did not have complete audiogram results, i.e., patients who were missing data for 250 Hz, 500 Hz, 1000 Hz, 2000 Hz, or 4000 Hz, and patients whose audiogram was not performed in air (e.g., through bone or a cochlear implant). Due to small sample sizes, i.e., less than 10 patients in a demographic group for one or more severity levels, we excluded patients who were coded in AudGenDB as being Black Hispanic/Latino, Asian Hispanic/Latino, Native (which included American Indian, Alaska Native, Native Hawaiian, or Other Pacific Islander), "Other", or "Multiple Races". From the remaining data, we ensured an even sampling of patients coded as Male and Female (112 of each) and patients coded across the four remaining race and ethnicity groups: White non-Hispanic/Latino, White Hispanic/Latino, Black non-Hispanic/Latino, and Asian non-Hispanic/Latino (56 of each). For each hearing loss severity group, the mean ages were normal: 10.90 ± 0.64 years, slight: 10.07 ± 0.62 years, mild: 9.72 ± 0.74 years, moderate: 10.41 ± 0.80 years, moderately severe: 10.04 ± 0.66 years, severe: 10.53 ± 0.80 years, and profound: 9.69 ± 0.81 years. The patient audiograms were converted into the node performance factors, b_i, using a fitting tool in UR_EAR [**17,1**]. To test the association between the resulting cochlea graph features and hearing loss severity, the seven hearing loss levels were represented by numbers one through seven. The Pearson correlation coefficient was then calculated between the hearing loss level variable and each graph feature. The patient with profound hearing loss whose cochlea graph was chosen to perform graph Fourier transform filtering had the following audiogram: 85 dB at 250 Hz, 95 dB at 500 Hz, 100 dB at 1 kHz, 100 dB at 2 kHz, 110 dB at 4 kHz, 90 dB at 8 kHz.

SI References

1. M. S. Zilany, I. C. Bruce, L. H. Carney, Updated parameters and expanded simulation options for a model of the auditory periphery. *The Journal of the Acoustical Society of America*, **135**, 283-286 (2014). https://doi.org/10.1121/1.4837815

2. J. A. Germiller *et al.,* AudGenDB: A Public, Internet-Based, Audiologic/Otologic/Genetic Database

for Pediatric Hearing Research. *Otolaryngology–Head and Neck Surgery*, **145**, P235-P236 (2011). https://doi.org/10.1177/0194599811415823a329

3. G. Mateos, S. Segarra, A. G. Marques, A. Ribeiro, Connecting the dots: Identifying network structure via graph signal processing. *IEEE Signal Processing Magazine*, **36**, 16-43 (2019). https://doi.org/10.1109/MSP.2018.2890143

4. V. Kalofolias, “How to learn a graph from smooth signals” in *Proceedings of the 19th International Conference on Artificial Intelligence and Statistics*, A. Gretton, C. C. Robert, Eds. (Proceedings of Machine Learning Research, 2016), pp. 920-929. https://proceedings.mlr.press/v51/kalofolias16.html

5. N. Perraudin *et al.*, GSPBOX: A toolbox for signal processing on graphs. arXiv [Preprint] (2016). https://arxiv.org/abs/1408.5781 (accessed 7 February 2022).

6. N. Perraudin *et al.,* The Graph Signal Processing Toolbox. GitHub. https://epfl-lts2.github.io/gspbox-html/doc/ (accessed 7 February 2022).

7. M. Pietsch et al., Spiral form of the human cochlea results from spatial constraints. *Scientific reports*, **7**, 7500 (2017). https://doi.org/10.1038/s41598-017-07795-4

8. D. D. Greenwood, A cochlear frequency-position function for several species—29 years later. *The Journal of the Acoustical Society of America*, **87**, 2592-2605 (1990). https://doi.org/10.1121/1.399052

9. G. Burwood, P. Hakizimana, A. L. Nuttall, A. Fridberger, Best frequencies and temporal delays are similar across the low-frequency regions of the guinea pig cochlea. *Science Advances*, **8**, eabq2773 (2022). https://doi.org/10.1126/sciadv.abq2773

10. C. A. Shera, J. J. Guinan Jr, A. J. Oxenham, Revised estimates of human cochlear tuning from otoacoustic and behavioral measurements. *Proceedings of the National Academy of Sciences*, **99**, 3318-3323 (2002). https://doi.org/10.1073/pnas.032675099

11. A. Hagberg, D. Schult, P. Swart, NetworkX. GitHub. https://github.com/networkx Deposited 5 March 2022.

12. M. E. Newman, Modularity and community structure in networks. *Proc. Natl. Acad. Sci. U.S.A.* **103**, 8577–8582 (2006). https://doi.org/10.1073/pnas.0601602103

13. Z. Zuo, Python modularity maximization. GitHub. https://zhiyzuo.github.io/python-modularity-maximization/ Deposited 23 February 2018.

14. M. E. Bonomo, C. Karmonik, A. K. Brandt, J. T. Frazier, Modularity allows classification of human brain networks during music and speech perception. arXiv [Preprint] (2020) https://arxiv.org/abs/2009.10308 (accessed 19 July 2022).

15. L. Fritts *et al.*, Data from “Musical Instrument Samples.” University of Iowa. Available at https://theremin.music.uiowa.edu/MIS.html. Deposited August 2014.

16. J. Hillenbrand, R. A. Houde, Vowel recognition: Formants, spectral peaks, and spectral shape. *The Journal of the Acoustical Society of America*, **98**, 2949-2949 (1995). https://doi.org/10.1121/1.414088

17. I. C. Bruce, Y. Erfani, M. S. Zilany, A phenomenological model of the synapse between the inner hair cell and auditory nerve: Implications of limited neurotransmitter release sites. *Hearing research*, **360**, 40-54 (2018). https://doi.org/10.1016/j.heares.2017.12.016
